# Validating clinical characteristics of primary failure of eruption (PFE) associated with *PTH1R* variants

**DOI:** 10.1186/s40510-021-00387-z

**Published:** 2021-12-13

**Authors:** Cristina Grippaudo, Isabella D’Apolito, Concetta Cafiero, Agnese Re, Pietro Chiurazzi, Sylvia A. Frazier-Bowers

**Affiliations:** 1 School of Dentistry, Università Cattolica del Sacro Cuore, L.go Agostino Gemelli 8, 00168 Rome, Italy; 2 Fondazione Policlinico Universitario “A. Gemelli” IRCCS, L.go Agostino Gemelli 8, 00168 Rome, Italy; 3 Medical Oncology, S.G. Moscati, 74010 Statte, Taranto Italy; 4 Università Cattolica del Sacro Cuore, 00168 Rome, Italy; 5 Dipartimento Universitario Scienze della Vita e Sanità Pubblica, Sezione di Medicina Genomica, Università Cattolica del Sacro Cuore, Largo Francesco Vito 1, 00168 Rome, Italy; 6 UOC Genetica Medica, Fondazione Policlinico Universitario “A. Gemelli” IRCCS, Largo Agostino Gemelli 8, 00168 Rome, Italy; 7 UNC Adams School of Dentistry, Chapel Hill, NC 27599 USA

**Keywords:** Primary failure of eruption, Orthodontics, *PTH1R* gene, Dental eruption, PFE diagnosis

## Abstract

**Background:**

Primary failure of eruption (PFE) is a hereditary condition, and linkage with variants in the *PTH1R* gene has been demonstrated in many cases. The clinical severity and expression of PFE is variable, and the genotype–phenotype correlation remains elusive. Further, the similarity between some eruption disorders that are not associated with *PTH1R* alterations is striking. To better understand the genotype–phenotype correlation, we examined the relationship between the eruption phenotype and *PTH1R* genotype in 44 patients with suspected PFE and 27 unaffected relatives. Sanger sequencing was employed to analyze carefully selected PFE patients. Potential pathogenicity of variants was evaluated against multiple genetic databases for function prediction and frequency information.

**Results:**

Mutational analysis of the *PTH1R* coding sequence revealed 14 different variants in 38 individuals (30 patients and 8 first-degree relatives), 9 exonic and 5 intronic. Their pathogenicity has been reported and compared with the number and severity of clinical signs. In 72.7% of patients with pathogenic variants, five clinical and radiographic criteria have been found: involvement of posterior teeth, involvement of the distal teeth to the most mesial affected, supracrestal presentation, altered vertical growth of the alveolar process and posterior open-bite. In cases with mixed dentition (3), the deciduous molars of the affected quadrant were infraoccluded.

**Discussion:**

The probability of an affected patient having a *PTH1R* variant is greater when five specific clinical characteristics are present. The likelihood of an eruption defect in the absence of specific clinical characteristics is rarely associated with a *PTH1R* mutation.

**Conclusions:**

We report here that systematic clinical and radiographic observation using a diagnostic rubric is highly valuable in confirming PFE and offers a reliable alternative for accurate diagnosis.

## Background

Tooth eruption is a complex process that represents an integral part of the broader tooth developmental process [1]. It is known that the tooth follicle interacts with both osteoblasts and osteoclasts during the tooth eruption process [2, 3]. To date, however, neither the eruption mechanism nor the factors controlling eruption are completely understood [4, 5].

Eruption disorders are ideally classified based on their etiology; those secondary to obstruction (cysts, ankyloses, lateral tongue pressure, impaction, etc.), or those with genetic underpinnings (e.g., primary failure of eruption (PFE); cleidocranial dysplasia, Hunter’s disease and osteopetrosis) should be considered [6].

Among the more common diagnostic distinctions, mechanical failure of eruption (MFE), ankylosis and primary failure of eruption (PFE) are examples of non-syndromic eruption disorders, but with vastly different clinical implications. MFE is defined as eruption failure due to mechanical obstruction of the eruption pathway, which can be treated successfully with the removal of the obstruction. Ankylosis is defined as the fusion of cementum to alveolar bone, but the practical diagnosis of ankylosis relies on the clinical appearance of infraocclusion and the absence of a periodontal ligament in radiographs [6–8]. In case of an early diagnosis of a first molar’s ankylosis, the therapy consists in the extraction of the ankylosed tooth and probably the second molar will drift mesially and will erupt [9].

Primary failure of eruption (OMIM #125350) was first described by Proffit and Vig [10] as a condition in which non-ankylosed teeth fail to erupt. Involved teeth can erupt partially and then cease to erupt, becoming relatively submerged although not being ankylosed. Posterior teeth are also affected in PFE, resulting in a posterior open bite.

Diagnosis of PFE is extremely important to avoid the consequences of employing a continuous archwire [11]. Indeed, PFE-affected teeth have little or no response to orthodontic treatment with a tendency to encounter ankylosis or intrude adjacent teeth [9].

These broad categories of dental eruption failure can be distinguished based on the following diagnostic parameters: (1) patient dental history; (2) clinical and phenotypic characterization and; (3) genetic characterization. The well-documented patient dental history is critical and allows understanding the developmental trajectory through serial photographs and radiographs of the eruption sequence. For example, evaluation of the patient natural history is relevant for identifying or excluding MFE based on the identification of other pathology that may have caused a mechanical obstruction during a prescribed time period. Characteristic causes of MFE include dental crowding, the presence of cysts, and pathology resultant from trauma. In the absence of detailed clinical documentation over time, it is difficult to rule out mechanical obstruction as the cause for eruption failure. PFE can be distinguished from MFE and other eruption disorders by the use of a diagnostic rubric that provides clinical and phenotypic characterization [12, 13]. As with most causes of eruption failure, PFE can present in a unilateral or symmetrically bilateral pattern, with one or all posterior quadrants involved. Of the two described types of PFE, Type II can be easily mistaken for ankylosis as the second molar, by definition, shows more eruption that the first molar [11, 12, 14]. Another key characteristic of PFE in general is the abnormal or complete lack of response to orthodontic force, so that affected teeth cannot be moved into their proper position [10]. Despite the expanding base of knowledge describing the eruption disorders, there is a gap in the knowledge of the tooth eruption mechanism.

Although the presence of specific clinical signs allows suspicion of PFE, there is wide variability of phenotypic expression and the diagnosis can be established by sequencing the parathyroid hormone 1 receptor gene (*PTH1R*) [OMIM #168468]. Ongoing genetic characterization of PFE is largely associated with autosomal dominant loss-of-function variants in the *PTH1R* gene, located on chromosome 3p21-p22.1, causing haploinsufficiency of the receptor [6, 10, 15–19]. Variants in *PTH1R* have also been associated with Jansen chondrodysplasia (OMIM #156400), Blomstrand chondrodysplasia (OMIM #215045), Eiken syndrome (OMIM #600002) and Ollier enchondromatosis (OMIM #166000). These conditions are characterized by abnormal skeletal development, and they are due either to recessive loss-of-function variants or a dominant gain-of-function variant in the *PTH1R* gene [20–23].

While it is helpful to analyze the *PTH1R* gene prior to treatment planning [15], merely establishing a family history through interviews and detailed clinical records on multiple family members is quite powerful. When analysis of the *PTH1R* gene is available, it represents an early diagnosis that is independent of family history [6, 10, 25]. In rare cases *PTH1R* variants may be associated with incomplete penetrance [24, 26], further complicating the genotype–phenotype correlation. These and other deficiencies underscore the need to improve the gaps of knowledge and to explore the characteristics of an informative cohort.

Several studies in the literature have elevated the finding that specific clinical characteristics are correlated with functional alterations in the *PTH1R* gene [10, 12–19]. In our report, we interrogate and validate that specific clinical characteristics [12, 13] correlate with the presence of *PTH1R* variants in a cohort of patients with suspected PFE and their relatives.

## Methods

### Clinical analysis

44 patients (26 males and 18 females), 23 in permanent dentition (age range 12–45 years) and 21 in mixed dentition (age range 5–12 years) with eruption disorders were enrolled in the Dental Clinic of the Policlinico “A. Gemelli” in Rome from November 2015 to May 2019. The study protocol was prepared in accordance with the Declaration of Helsinki. Ethical approval was obtained from the Ethics Committee of the Catholic University of Sacred Heart of Rome (study ID 565—11/2015). Patients with suspected PFE were evaluated by three qualified orthodontists including clinical examination, intraoral photographs and orthopanoramic radiography. Assessment of clinical signs was established with the consent of all three examiners, on a case-by-case basis (see Tables 3, 4, 5 for included characteristics). Patients were included in the study if at least one infraoccluded permanent molar was reported in permanent dentition, or one or more infraoccluded deciduous molar were observed in mixed dentition.

All enrolled patients were examined clinically and provided a saliva sample after consenting to study participation. DNA was extracted from saliva and subjected to PCR and sequencing as described below. Our analysis included 27 unaffected relatives of affected individuals.

### PTH1R sequencing

DNA was collected using a cytobrush (Cooper Surgical, Trumbull, CT, USA) and extracted and purified with the 401 Genomic DNA Tissue Kit (MagCore®, RBC Bioscience Corp.). Amplification of all coding exons of *PTH1R* [AB 3500 Genetic Analyzer (Life Technologies, Carlsbad, CA, USA)] was carried out following quantification with the Qubit Fluorometer (Life Technologies, Carlsbad, CA, USA) and verification of DNA integrity with agarose gel electrophoresis as previously described [25]. Primers employed for sequencing the coding sequence and exon–intron junctions of the *PTH1R* gene are reported in Table 1.

**Table 1:** Primers employed for sequencing *PTH1R* coding exons

*PTH1R* exon	Primer sequence
3 FORWARD	5′-AGCCTGACGCAGCTCTGCA-3′
3 REVERSE	5′-CCCACAGTCCAGACATCCCA-3′
4 FORWARD	5′-AGAGCAGATTCCCCACATGC-3′
4 REVERSE	5′-TTCACCTGGCTCTGTATCCT-3′
5 FORWARD	5′-TCCTCACCCATCGTCTCAGAT-3′
5 REVERSE	5′-AAGAGCCAAGAAGCATGAGC-3′
6 FORWARD	5′-AGATGTATTCATCCTTCTGGG-3′
7 REVERSE	5′-TAAGGTTGCTGGAGGAGTCAAG-3′
8 FORWARD	5′-AAATTCACTCCCACCCCACG-3′
8 REVERSE	5′-TGGACAGGAAGCTGGGTTGT-3′
9 FORWARD	5′-ACAACCCAGCTTCCTGTCCA-3′
9 REVERSE	5′-GTTGCGAGGGACCCTATAAG-3′
10 FORWARD	5′-AAACGAAGCCTGCCCCTTC-3′
10 REVERSE	5′-GCCTGGAATAGGGTCAGGAT-3′
11 FORWARD	5′-GGAATGACCTTGTGGACAGC-3′
11 REVERSE	5′-TAGCTGTTGAGGACACAGGG-3′
12 FORWARD	5′-AGGGTCACAGGAGGCTACTT-3′
12 REVERSE	5′-TGTCACTGCATCTCTGGGTG-3′
13 FORWARD	5′-CCAGCCCAGAAAGGAAAACC-3′
13 REVERSE	5′-TAGTGCAGGGCCTGGTACAA-3′
14 FORWARD	5′-AGGTGAACTGGGTTGTCCTC-3′
15 REVERSE	5′-GAATGTCCTCAGGGGTGTTC-3′
16 FORWARD	5′-CACTTGGCCTTGGAGTTTCC-3′
16 REVERSE	5′-CCACCCATCTTTTGGTCC-3′

### Analysis of variants

All variants have been positioned on the GRCh37/hg19 human genome assembly, and their frequency in the general population was retrieved from the Genome Aggregation Database (GnomAD) [27] and the Single Nucleotide Polymorphism Database (dbSNP) [28]. In order to evaluate the potential pathogenicity of *PTH1R* variants, we looked them up in the NCBI ClinVar database [29] and in the Leiden Open Variation database [30]. Finally, we cross-checked each variant using VarSome [31], a powerful annotation tool and search engine for human genomic variants [32].

### Statistical analysis

Descriptive statistics were calculated. The frequency of PFE key clinical traits was analyzed using Graph Pad Prism program (https://www.graphpad.com/scientific-software/prism/). Fisher’s exact test was employed to evaluate the significant association between variables, i.e., group with *PTH1R* (likely) pathogenic variants, group with *PTH1R* benign variants and group without *PTH1R* variants.

## Results

### Genetic results

Molecular analysis of the *PTH1R* coding sequence revealed 14 different variants in 38 affected individuals (27 patients and 11 first-degree relatives). Table 2 summarizes all variants found in our cohort (9 exonic and 5 intronic) along with their relative frequency in heterozygous and homozygous subjects from the general population as reported in the Genome Aggregation Database. The 9 exonic *PTH1R* variants had different effects on the protein structure: 1 nonsense variant, 1 frameshift variant and 4 missense variants, all not reported in the control population; 3 synonymous variants, two of which are reported in GnomAD. The 5 intronic variants have no obvious effect on splicing and all but one are very frequent in normal control individuals.

**Table 2:**
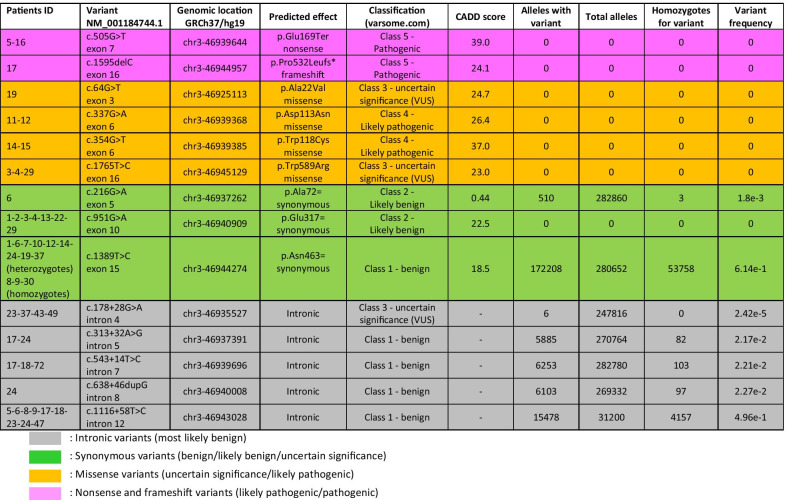
*PTH1R* variants found in our cohort with their cDNA and genomic location, predicted effect and classification, CADD scores for exonic variants, and frequency in gnomAD

The nonsense variant, c.505G>T—p. (Glu169Ter), not reported in any genomic databases, was identified in patient 5 and his mother (ID.16), who has no clinical signs of PFE. This variant introduces a premature stop codon early in the coding sequence and is classified as Class 5 (pathogenic), possibly resulting in nonsense-mediated decay and complete absence of the protein.

The frameshift variant, c.1595delC—p.(Pro532Leufs*), due to the deletion of one cytosine in a short homopolymeric tract (1593–1595), results in the production of a longer protein with 21 extra amino acids at the C-terminus and is classified as Class 4 (likely pathogenic).

Two of the 4 missense variants were previously reported [24], namely c.64G>T—p.(Ala22Val) and c.1765T>C—p.(Trp589Arg), and are classified as variants of unknown significance (VUS/Class 3). The c.64G>T causes the replacement of alanine in position 22 with a valine in the extracellular domain of the protein and was found in a very young patient (ID.19) with mixed dentition. The c.1765T>C was found in two sibs and their mother (ID. 3-4-29). This variant replaces tryptophan 589 with arginine at the end of the protein in the intracellular domain of the receptor.

Two other missense variants, c.337G>A—p.(Asp113Asn) and c.354G>T—p.(Trp118Cys), have never been previously reported and are both classified as likely pathogenic (Class 4). The c.337G>A results in aspartic acid to asparagine change in position 113 in the extracellular domain of the protein, and was found in the proband (ID. 11) and in his mother (ID. 12). The c.354G>T, resulting in the substitution of tryptophan 118 with cysteine, was detected in two brothers (ID. 14-15), whose parents were unavailable for clinical examination and genetic testing.

Only one of the synonymous variants found in our patients, c.951G>A—p.(Glu317=), was absent in the control population (Class 3, uncertain significance); interestingly this variant was also found in the two sibs and mother with the c.1765T>C variant described above. Furthermore, the c.951G>A was also found in a bilateral Type I PFE (ID. 2), in a unilateral Type 2 PFE (ID. 1), in a patient with failed eruption of upper and lower second molars (ID. 13), while the last patient (ID. 22) had a severe left open bite involving premolars and an included upper left canine.

Although the second synonymous variant, c.216G>A—p.(Ala72=), is reported in GnomAD with a frequency of 1.8 × 10^–3^, suggesting its probable benignity, we found it in one patient (ID. 6) with bilateral Type I PFE.

The last synonymous variant, c.1389T>C—p.(Asn463=), has an extremely high frequency and is reliably classified as benign.

All intronic variants found in our cohort do not change the splicing donor (GT) nor the acceptor (AG) sites and are unlikely to alter mRNA splicing. Four out of 5 of the identified intronic variants are indeed quite frequent in the control population and can be considered benign (Class 1) common polymorphisms. Only the c.178 + 28G>A, found in intron 4, has a very low frequency (2.42 × 10^–5^) and is considered a VUS (Class 3), as indicated in Table 2.

### Clinical and radiographic findings

Genetic analysis allowed us to divide our patients into three groups: 11 patients (6 male and 5 female), in whom heterozygous nonsense, frameshift and missense *PTH1R* variants were found; 19 patients (12 male and 7 female) with synonymous and intronic variants, and 17 patients (8 male and 9 female) without *PTH1R* variants. In Tables 3, 4 and 5, we categorized patients on the basis of their clinical and radiographic findings in order to highlight the genotype–phenotype correlation.

**Table 3:** Clinical signs in patients with *PTH1R* variants that are likely pathogenic (nonsense, frameshift and missense)

Patient ID	Age and dentition	1. *permanent molars are affected*	2. *teeth distal to a PFE-affected molar are also affected*	3. *supracrestal presentation (eruption pathway completely clear)*	4. *there is a reduction of vertical growth of the alveolar bone in the affected regions*	5. *a lateral open bite is present in the affected regions*	6. *deciduous infraoccluded in mixed dentition*	7. asymmetry due to bilaterally unbalanced eruption of the teeth	8. affected individual presents with a bi-lateral affection of PFE	9. affected molars are located in the basal bone of the jaws	10. affected molars show dilacerated roots
3*	8 mix	x	x	x	x	x	x		x		x
4*	17 perm	x	x	x	x	x		x	x	x	x
29	45 perm	x		x					x		
5*	22 perm	x	x	x	x	x			x	x	
16	45 perm										
12*	14 perm	x	x	x	x	x		x			
11*	43 perm	x	x	x	x	x		x	x		
14*	15 perm	x	x	x	x	x		x		x	
15*	12 perm	x	x	x	x	x		x		x	
17*	6 mix	x	x	x		x	x		x		
19	7 mix			x			x deciduous only	x			

The hallmark signs (key PFE traits) are italics and listed first, from 1 to 6

Patients marked by * showed at least 5 hallmark signs

**Table 4:** Clinical signs in patients with *PTH1R* variants that are likely benign (synonymous and intronic)

Patient ID	Age and dentition	1. *permanent molars are most frequently affected*	2. *teeth distal to a PFE-affected molar are also affected*	3. *supracrestal presentation (eruption pathway completely clear)*	4. *there is a reduction of vertical growth of the alveolar bone in the affected regions*	5. *a lateral open bite is present in the affected regions*	6. *deciduous infraoccluded in mixed dentition*	7. asymmetry due to bilaterally unbalanced eruption of the teeth	8. affected individual presents with a bi-lateral affection of PFE	9. affected molars are located in the basal bone of the jaws	10. affected molars show dilacerated roots
1	18 perm	x		x		x		x			
2*	8 mix	x	x	x		x	x		x		x
6	8 mix	x	x	x		x			x		x
7	8 mix	x		x	x	x	x				x
8*	16 mix	x	x	x		x	x	x			x
9	8 mix	x		x	x	x		x		x	
10*	17 perm	x	x	x	x	x		x	x	x	
13	12 perm	x		x					x	x	
18	6 mix	x		x		x	x	x	x		
22	17 perm	x		x		x		x			
24	11 mix	x		x		x	x		x		
30	14 perm	x		x				x			
72	12 perm	x	x	x	x			x		x	x
20	12 mix	x		x							
23	15 perm	x		x				x		x	
37	8 mix			x		x	x dec. only				
43	12 perm			x							
47	7 mix			x			x dec. only	x			
49	10 mix			x			x dec. only				

The hallmark signs (key PFE traits) are italics and listed first, from 1 to 6

Patients marked by * showed at least 5 hallmark signs

**Table 5:** Clinical signs in patients without *PTH1R* variants

Patient ID	Age and dentition	1. *permanent molars are most frequently affected*	2. *teeth distal to a PFE-affected molar are also affected*	3.* supracrestal presentation (eruption pathway completely clear)*	4.* there is a reduction of vertical growth of the alveolar bone in the affected regions*	5.* a lateral open bite is present in the affected regions*	6.* deciduous infraoccluded in mixed dentition*	7. asymmetry due to bilaterally unbalanced eruption of the teeth	8. affected individual presents with a bi-lateral affection of PFE	9. affected molars are located in the basal bone of the jaws	10. affected molars show dilacerated roots
39	5,3 mix						x	x			
40	6,1 mix						x	x			
41	8 mix						x	x			
42	13 perm	x						x			
62	13 mix										
44	10,5 mix						x	x			
45	15 perm	x		x	x			x			
52	13 mix						x	x			
50	6,3 mix						x	x			
55	8 mix						x		x		
71*	19 perm	x	x	x	x	x		x		x	
64	14 mix						x	x			
65	8,8 mix						x		x		
36	13 perm	x		x				x			
31	14 perm	x		x				x			
35	14 perm	x		x				x			
51	13 perm	x		x		x		x			

The hallmark signs (key PFE traits) are italics and listed first, from 1 to 6

Patients marked by * showed at least 5 hallmark signs

**Fig. 1:**
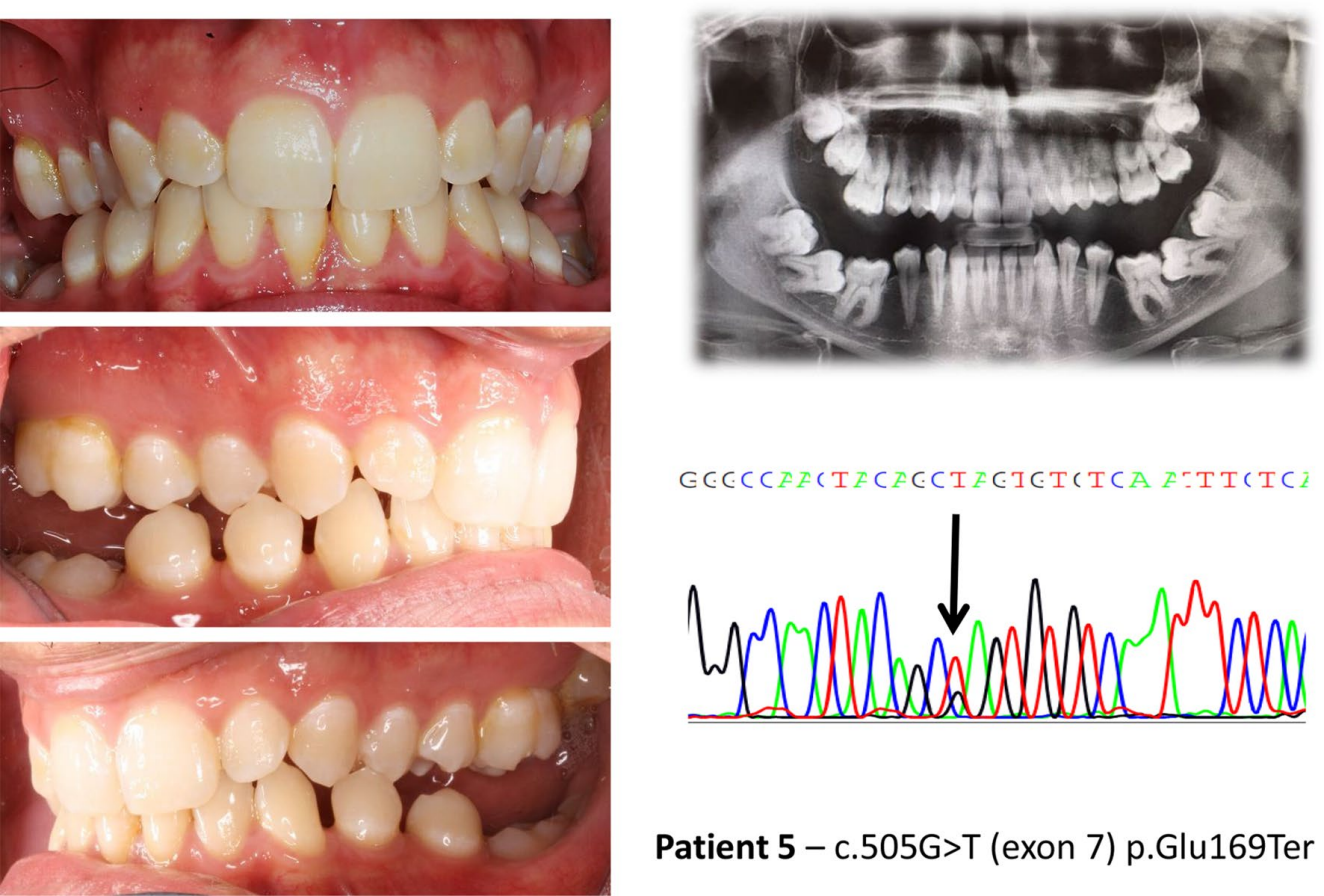
Clinical features and genetic findings of patient 5 with PFE and a *PTH1R* nonsense variant

In patients with *PTH1R* variants (nonsense, frameshift and missense) that alter the protein structure (Table 3), the open bite is more severe and Type I PFE is frequent. In this group of patients, 8 out of 11 (72,7%) showed all the typical signs of PFE reported by Pilz et al. [13] in 100% of their patients with pathogenic *PTH1R* variants (Fig. 1). One patient (ID. 19), carrying the missense variant c.64G>T, had a very mild phenotype at 8 years of age but was lost to follow-up for further characterization. Our clinical characterization also revealed a disconnect in the eruption phenotype among 3 parent–child pairs carrying the same *PTH1R* change. While three mothers of children with clinical signs of PFE also carried the variant, their corresponding phenotype varied. One mother had a normal phenotype, the second had a unilateral open bite (corrected by prosthetic crowns) and the third showed the inclusion of an upper canine and a lower second premolar (not typical of PFE). Notably the relative infraocclusion of the molar teeth was insignificant.

**Fig. 2:**
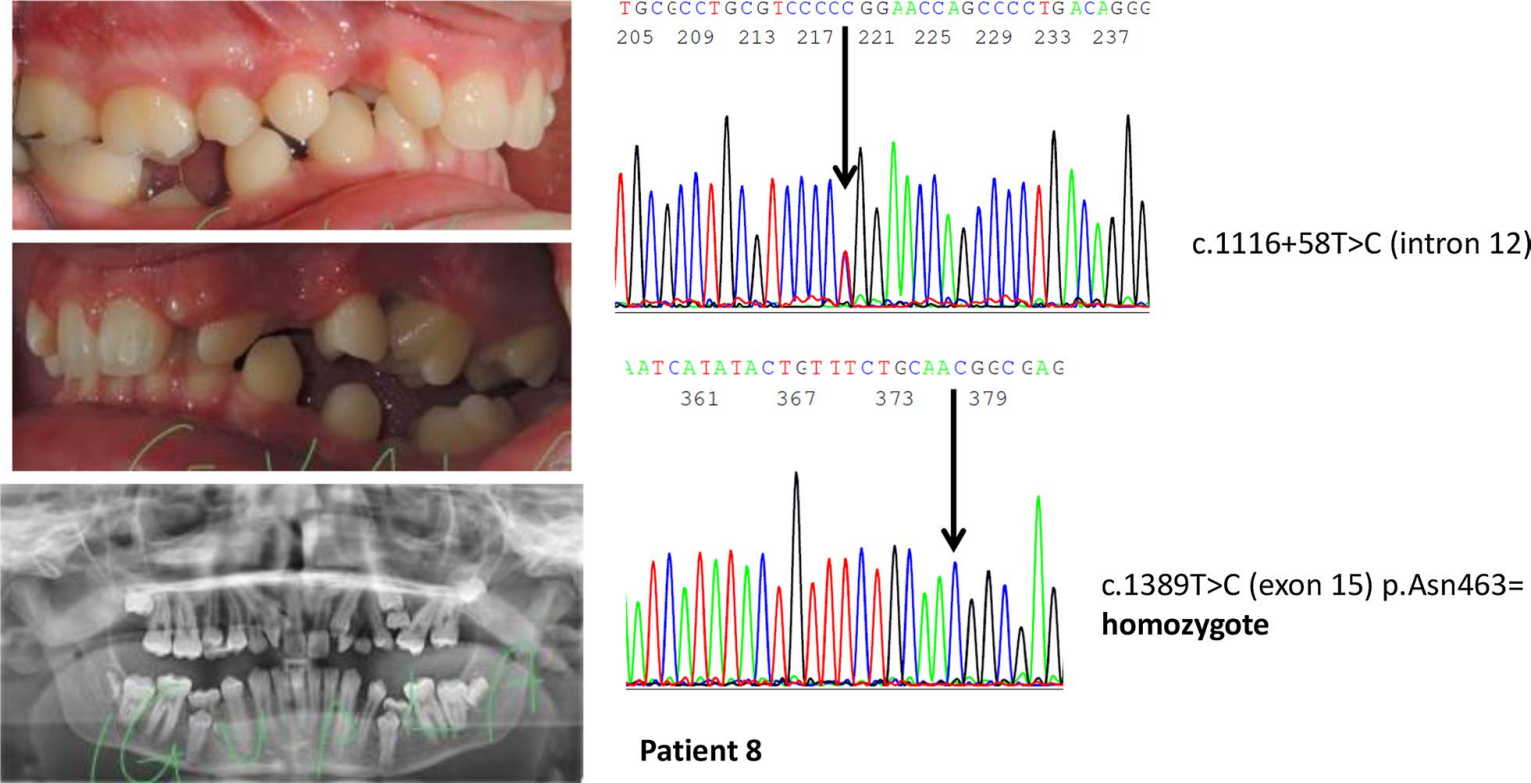
Clinical features and genetic findings of patient 8 with PFE and both a synonymous and an intronic *PTH1R* variant

**Fig. 3:**
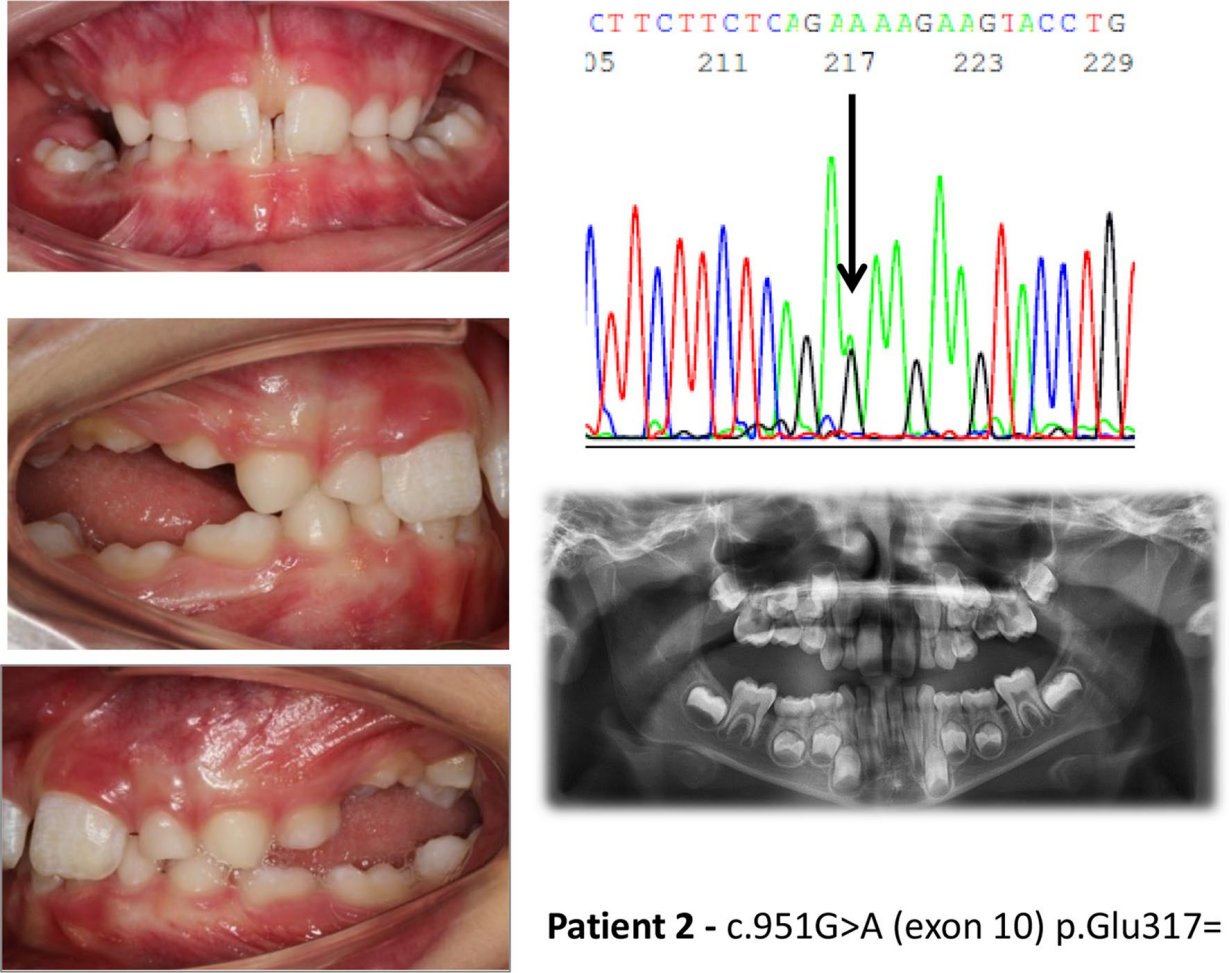
Clinical features and genetic findings of patient 2 with PFE and a synonymous *PTH1R* variant

**Fig. 4:**
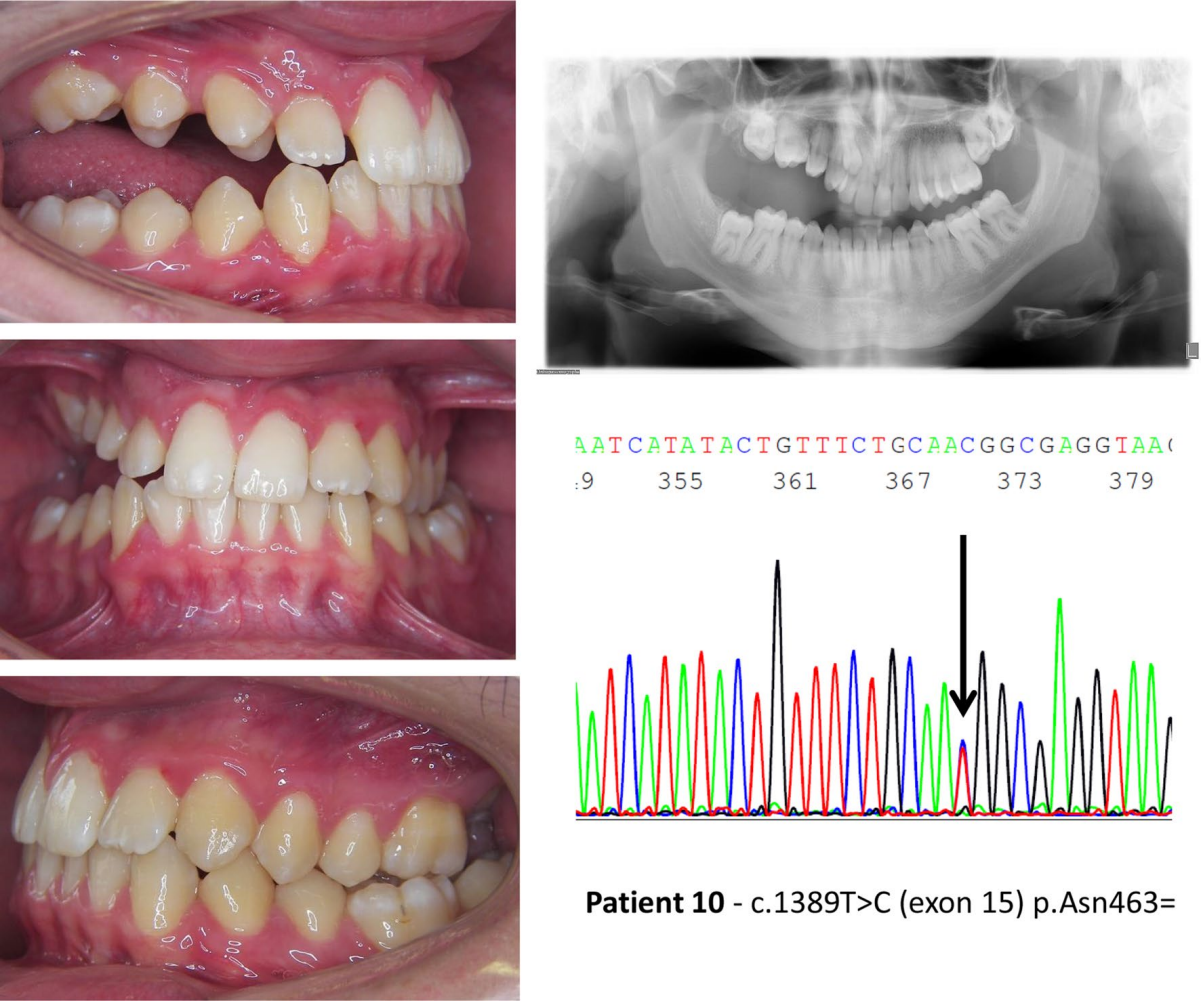
Clinical features and genetic findings of patient 10 with PFE and an intronic *PTH1R* variant

**Fig. 5:**
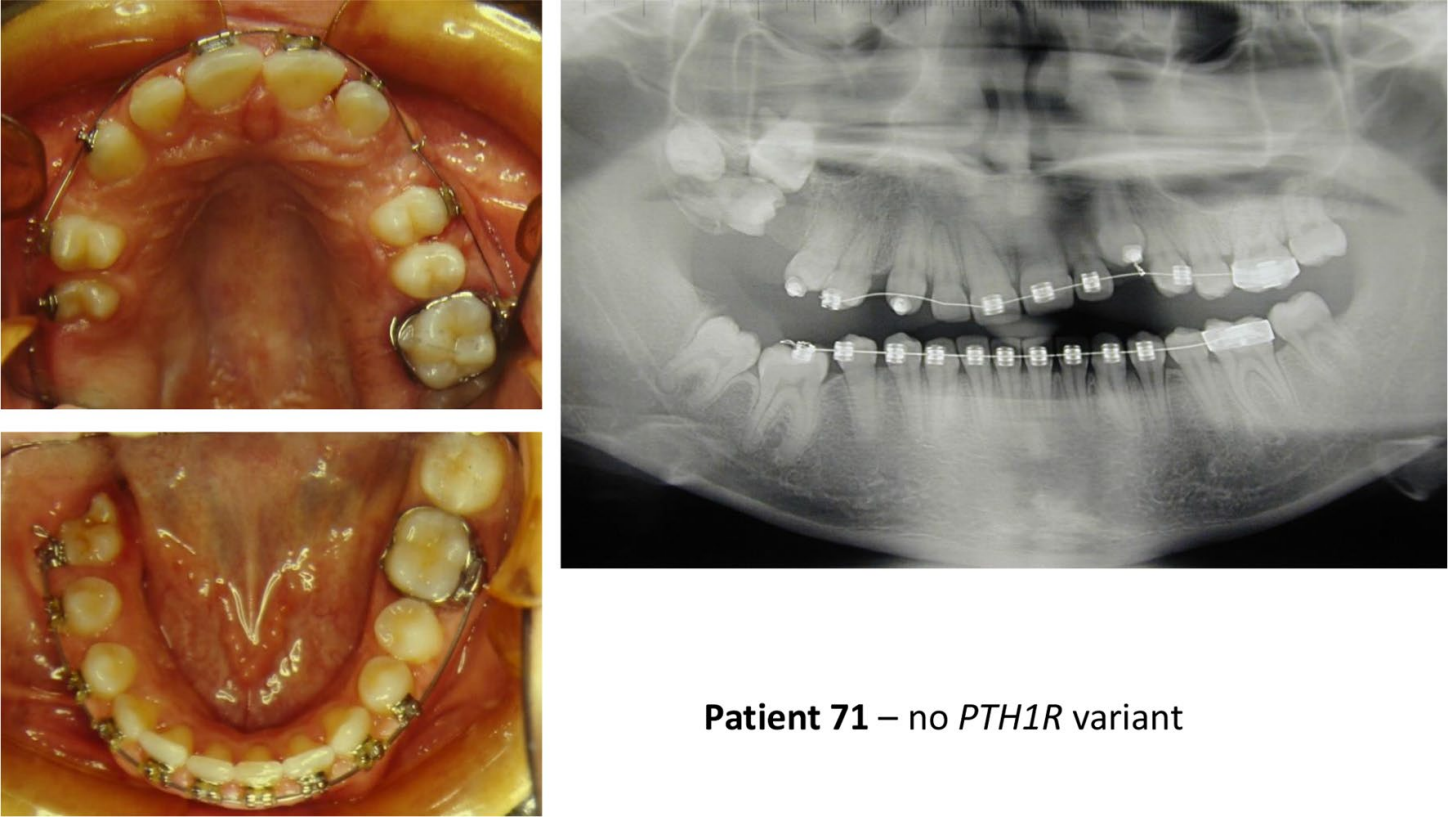
Clinical features and genetic findings of patient 71 with PFE but without *PTH1R* variants

In patients with synonymous and intronic *PTH1R* variants (Table 4), whose functional effect is uncertain, only three out of 19 had typical PFE. In these three patients, we noticed the presence of all the most typical signs of PFE. One was very atypical, with premolar inclusions, but the other two looked as typical PFE (Figs. 2, 3, 4). In the third group (Table 5), without *PTH1R* variants, only one out of 17 had all typical PFE signs. The diagnosis based on clinical results eventually allowed us to exclude the diagnosis of PFE, and the results of the genetic test confirmed it. The only patient of this group who had a severe clinical phenotype, showed the first upper right molar infraoccluded and the second and third molars included, and a history of orthodontic therapy failure (Fig. 5).

### Statistical results

**Fig. 6:**
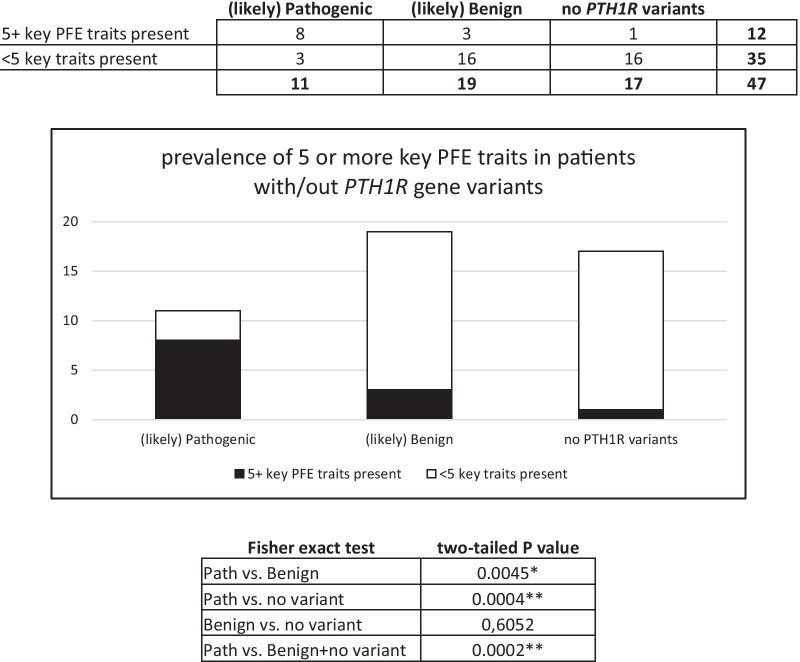
Statistical analysis and significant association of 5 or more PFE key traits (hallmark signs) with (likely) pathogenic *PTH1R* variants

As indicated in Fig. 6, from a total of 11 subjects with pathogenic *PTH1R* variants, 5 or more clinical hallmarks (key traits) of PFE were present in 8 of them (72.7%). Among the group of 19 subjects with benign *PTH1R* variants, 5 or more hallmarks were observed in 3 (15.8%), while in the group without variants (17 subjects) only 1 showed all 5 key traits (5.9%). Fisher’s exact test showed a significant association between the presence of the key clinical traits in the group with pathogenic variants compared to the group with benign variants (*p* = 0.0045), and to the group without variants (*p* = 0.0004), and to the groups with benign variants and without variants pooled together (*p* = 0.0002). The comparison between the group with benign variants versus the group without *PTH1R* variants showed no significant difference (*p* = 0.6052), as expected.

## Discussion

A total of 44 patients and 3 mothers were sequenced in this study, of which 30 were genetically identified as carriers of variants of the *PTH1R* gene, while in the other 17 no variants were found. The variant carrier group included 18 males and 12 females, although PFE cases are not known to have a gender difference [13]. The non-carrier group included 8 males and 9 females, again suggesting no gender difference. As detailed in Table 3, 11 of the 30 patients carrying pathogenic or likely pathogenic *PTHR1* variants had mostly bilateral Type I PFE, while asymmetrical involvement was more frequent in the 19 patients with benign or likely benign *PTH1R* variants. Molar root dilacerations were very rare, and the other clinical signs did not discriminate the pathogenicity of the *PTH1R* variants. The remaining 17 patients without *PTH1R* variants, with few notable exceptions, showed a less defined phenotype, sometimes limited to the involvement of a single dental element, when compared to *PTH1R*-positive patients.

Five clinical and radiographic criteria described by Pilz et al. [13] have been found in almost all carriers of *PTH1R* variants: involvement of posterior teeth, involvement of the distal teeth to the most mesial affected, supracrestal presentation (eruption pathway completely clear), altered vertical growth of the alveolar process and posterior open-bite. Statistical analysis (Fig. 6) showed a significant difference in the frequency of PFE key clinical traits between patients with (likely) pathogenic *PTH1R* variants and those with either benign or no variants. We therefore reject the null hypothesis of no association in favor of the alternative that a (non-zero) association exists in the population. In patients with pathogenetic variants of *PTH1R* and mixed dentition, one or more deciduous teeth were infraoccluded. Our data on deciduous dentition involvement are in agreement with Pilz et al. [13]. From the cohort reported here, we learned that systematic clinical and radiographic observations using a diagnostic rubric is highly valuable to validate the diagnosis of PFE. While genetic testing offers additional confirmation, toward confirming PFE, the rubric provided sufficient information for a correct diagnostic orientation, and for selecting patients eligible for genetic testing. However, a *PTH1R* variant was found only in 64% of PFE patients (30 of 47), and only 23% of these (11 out of 47) were carriers of pathogenic or likely pathogenic variants. The functional analysis of the effects of all *PTH1R* variants at the mRNA and protein level would be very useful, but it is challenging and has rarely been done [33–35]. We suspect that the phenotypic manifestation of PFE observed in the remaining patients might be due to variants in other genes, encoding other proteins involved in the complex process of tooth morphogenesis and eruption and not implicated in PFE pathogenesis yet [3–5]. The study of tooth morphogenesis, characterized by reciprocal epithelial–mesenchymal interactions, has benefited from the use of animal models, primarily mice [36, 37]. For example, Philbrick et al. [36] first showed that *PTH1R* is highly expressed in the alveolar bone surrounding the dental follicle, while its ligand PTHrP is produced by cells of the enamel epithelium and the stellate reticulum and is necessary for the reabsorption of the alveolar bone overlying the erupting tooth. Therefore, patients with typical PFE characteristics, who do not carry pathogenic variants in the *PTH1R* gene, could be recruited for further genetic studies that seek, for example, alterations in the Parathyroid Hormone Like Hormone (*PTHLH*) gene coding for Parathyroid hormone-related protein (PTHrP) [38]. Indeed, Philbrick et al. [36] elegantly demonstrated that, in the mouse, expression of PTHrP by the enamel epithelium is necessary for the reabsorption of the bone roof overlying the dental elements, while its absence determines PFE. Furthermore, Klopocki et al. [39] highlighted the role of PTHrP in human dental eruption, observing that two out of five families affected by E1 type brachydactyly (OMIM #113300) and carrying variants in the *PTHLH* gene, in addition to short stature and short fingers and metacarpals, also had multiple impacted teeth and/or PFE.

## Conclusion

Our findings on a large PFE cohort suggest that the key clinical traits of PFE are significantly more present in patients with pathogenic variants of the *PTH1R* gene. Additionally, our descriptive statistic suggests that a subject who have signs of PFE may not have variants of the *PTH1R* gene. Considering our data and those described in the literature, we believe it is useful, whenever possible, to carry out the genetic test. When clinical signs are present and testing is not possible, the risks of undertaking orthodontic therapy in a patient with a high probability of PFE should be carefully considered.

From our results, the diagnostic rubric allows to select patients with high possibility of PFE with a pathogenic *PTH1R* variant. Furthermore, if *PTH1R* variants are found, their pathogenic role should be questioned and at least an in silico analysis should be performed. If *PTH1R* variants are likely benign or are considered of uncertain significance, functional studies should be performed before linking any variant to the PFE phenotype. Finally, if *PTH1R* screening is negative and the alternative diagnoses of MFE or ankyloses have been excluded, we suggest to extend genetic testing at least to the *PTHLH* gene. In fact, considering the complex process of tooth formation and eruption and the multiple genes and corresponding proteins involved, the development of a dedicated gene panel would be highly desirable.

## Data Availability

The datasets used and/or analyzed during the current study are available from the corresponding author on reasonable request.
